# Exploring the reasons for women to engage in sex work in Tehran, Iran: A qualitative study

**DOI:** 10.1016/j.heliyon.2021.e08512

**Published:** 2021-11-29

**Authors:** Javad Yoosefi Lebni, Seyed Fahim Irandoost, Ali Akbar Dehghan, Arash Ziapour, Bahar Khosravi, Nafiul Mehedi

**Affiliations:** a Health Promotion Research Center, Iran University of Medical Sciences, Tehran, Iran; b Social Determinants of Health Research Center, Clinical Research Institute, Urmia University of Medical Sciences, Urmia, Iran; c Sociology, Shiraz University, Shiraz, Iran; d Social Development & Health Promotion Research Center, Health Institute, Kermanshah University of Medical Sciences, Kermanshah, Iran; e Students Research Committee, Kermanshah University of Medical Sciences, Kermanshah, Iran; f Department of Social Work, Shahjalal University of Science and Technology, Sylhet, Bangladesh

**Keywords:** Sex workers, Reasons, Conventional content analysis, Iran

## Abstract

**Background:**

Sex work is a growing phenomenon triggered by a number of causes. The current study uses a qualitative method to investigate the reasons why women participate in sex work in Tehran.

**Methods:**

The statistical population consisted of all female sex workers in Tehran, Iran, from which 22 individuals were chosen via snowball sampling. In-depth interviews were conducted for data gathering, and traditional content analysis was used for data analysis. Graneheim and Lundman's method was utilized to evaluate the data, and Guba and Lincoln's criteria were employed to determine the research's strength and transferability.

**Results:**

The results of the data analysis were divided into five categories (or themes) and 19 subcategories. The categories were familial instability, societal pressure, consumerism, social insecurity, and a meek and inefficient personality.

**Conclusion:**

Sex work may be avoided by raising women's social status, providing greater economic assistance, making society safer, particularly in workplaces, improving intra-family connections, boosting self-esteem and self-efficacy, and teaching them how to deal with life's issues.

## 1 Introduction

Sex work is a worldwide phenomenon [1], referring to the selling and purchasing of sexual services for a price [2, 3]. Sex labor is allowed in certain countries [4, 5], but it is prohibited and punished by law in most others, including Iran [6, 7, 8]. The growth in sex work rates continues unabated around the world, including in Iran [9, 10, 11]. The proportion of women who work as female sex workers worldwide ranges from 0.1% to 7.4% [12], with the overall number of female sex workers estimated to be 42 million women [13]. Because sex work is prohibited in Iran, there are no accurate figures, although it is believed that there are around 228,700 female sex workers in Iran [14].

Sex work is a high-risk activity, and the majority of female sex workers are vulnerable to sexually transmitted diseases (STIs) such as Hepatitis B and C [15, 16] and HIV through their customers and transmission to other sexual partners [17, 18, 19, 20]. In developing countries, about one-eighth of female sex workers are HIV-positive [21]. The rate of Infection among female sex workers in Iran is 4.5% [14]. Other issues that sex workers experience include vaginal infection, backache, stress, depression, and chronic fatigue [22, 23]. In addition to the physical health implications of sex labor, it also jeopardizes the mental health and financial stability of families and communities [7, 24].

One of the key concerns in scientific studies on prostitution is why women participate in sex work. According to research, sex work happens for a number of reasons, including economic difficulties [25, 26, 27], marital breakdowns [25, 28], immigration [26, 28, 29], and drug usage [30, 31, 32, 33].

Sex work in Iran began during the Qajar dynasty with the establishment of a brothel in Tehran called Qala-e-Shahr-e-Naw. Then, during the Pahlavi era, it got more intensive, and specific laws were enacted to govern the prostitutes' and their clients' behavior, such that over a thousand prostitutes worked there. When the Islamic Revolution broke out in 1978, prostitution was declared a felony, and the brothel was dismantled; most of the women who worked there were imprisoned, and several were killed [34]. Since sex work is prohibited in Iran, precise figures on the number of prostitutes are unavailable, but recent research indicates that sex work has grown in recent years [9, 10, 11]. Prostituted women in Iran are socially labeled, and the country's social and cultural context exposes the lives of prostituted women to abuse and violence [35, 36].

According to Allahqoli and Rahmani (2017), familial history, poor education, and societal hazards are the factors that predispose women to participate in sex work [10]. According to Karamouzian et al. (2016), women in Kerman turn to prostitution due to chronic vulnerability and poverty, restricted options, drug usage, and financial requirements [37].

In a qualitative research conducted in Hong Kong, Cheung et al. (2016) discovered that familial pressures, the need for money, and societal standards are the primary reasons that encourage women to engage in sex work [38]. In an Indian study, Karandikar (2013) discovered that poverty, being mistreated by one's husband, sexual abuse, and the loss of a parent or husband were key causes for engaging in sex work [39]. As a result, there are several routes and pressures that bring women into sex work, elements that are at the foundation of many of today's most serious social, psychological, political, and economic issues [40, 41].

Controlling sex work, like any other social and health issue, necessitates precise knowledge of all elements of it, particularly factors impacting the continual recruitment into the profession. Interviewing individuals who are affected by an issue is one approach to gaining a better understanding of why it occurs. Furthermore, because sex work is a felony in Iran and is done in secret, it is impossible to examine this phenomenon numerically, and most research done outside of Iran has been done quantitatively. As a result, this study took a qualitative approach, with the objective of identifying the reasons why women participate in sex work in Tehran, Iran.

## 2 Methods

### 2.1 Study design

The traditional content analysis method was utilized in the current investigation. In general, the qualitative approach is based on analytical and explanatory techniques, with the major emphasis on deep knowledge, complexity, intricacies, and context of the phenomena under investigation, requiring the researcher to be actively engaged in the research process. In traditional content analysis, interviewing participants provides a deeper understanding and larger data set of their experiences and viewpoints [42, 43]. The conventional qualitative content analysis was used since there was a lack of complete information and ideas concerning the causes of prostitution in Iran. Furthermore, researchers utilized conventional content analysis because they wanted to get straight and plain information from the study without imposing pre-determined ideas.

### 2.2 Setting and participants

The target population was comprised of all female sex workers living in Tehran, Iran, in 2019, of which 22 were chosen via snowball sampling. Previous sex job experience, willingness to engage in the study, and the capacity to answer questions were all inclusion criteria. Unwillingness to participate and leave the interview were exclusion criteria.

To that aim, after locating the first participant, she was invited to introduce herself, speak about her sex work experience, and offer the names of other sex workers who might be interested in joining. The majority of the participants were recruited in this manner, and the interviews were held in public settings such as parks or locations chosen by the participants. In reality, interviews were held in public areas, parks, and other convenient locations for participants, and coordination was done with the respondent to pick these surroundings, and if she was dissatisfied, another location for the interview was chosen based on her viewpoint.

### 2.3 Data collection

Semi-structured interviews were used to collect data over the spring and summer of 2019 (lasting 45 min). The interviews were done by a trained female assistant. The interview questions were developed through a review of the literature on sex work, with an emphasis on understanding the factors of this phenomenon, as well as consultation with the research team and specialists (Table 1).

**Table 1:** Checklist for questions from the interview guide.

No.	Questions
1	What motivated you to engage in sex work for the first time?
2	Were you previously familiar with sex work? If so, where did this familiarity emerge?
3	Were you motivated to do it on your own, or were you coerced into doing it?
4	Why did you not select another employment if you were dissatisfied with it?
5	What impact did your family have on influencing you to pursue sex work?
6	How do you evaluate the influence of society and your personality qualities on predisposing you to this sort of work?

Ethical approval was obtained from the Kermanshah University of Medical Sciences (IR.KUMS.REC.1398.393). The aims of the study were explained to the participants at the start of the interview sessions, and they were guaranteed confidentiality of their information and the preservation of their anonymity. All participants provided written informed consent. The entire sample population was calculated using the theoretical saturation criteria [44]. In other words, data collection was halted when the researchers determined that more interviews and observations would not provide them with new information. After obtaining consent from the participants, the interviews were taped using a voice recorder.

### 2.4 Data analysis

The first and second authors of the study carried out the data analysis utilizing Graneheim and Lundman's approach [45] and MAXQDA-ver. 2018. The researcher initially typed the interview transcript in the Microsoft Word 2017 program immediately following the first interview and on the same day, with the assistance of other research colleagues. The researchers carefully read the interview texts three times in the second stage to obtain a broad feel of them. In the third phase, all of the interview texts were carefully and methodically reviewed line by line to extract the initial codes. In the fourth phase, the researchers classified codes that had comparable meanings and concepts into one group and assessed their significance. The codes and categories were placed in the main categories in the fifth stage, which was more extensive and abstract in terms of ideas. Finally, the whole data analysis process was shared, and all of the article writers' perspectives were used in a joint session.

### 2.5 Trustworthiness

Lincoln and Guba's trustworthiness criteria were fulfilled to assess the quality of the results [46]. The researchers evaluated participant variety to boost the study's credibility. To ensure the research's dependability, the findings were made available to the participants so that they could voice their opinions, which were finally approved by all of them. Furthermore, the findings were forwarded to six famous qualitative researchers, who also approved the phases of analysis and findings. To improve confirmability while attempting to minimize researcher bias, all authors of the study were part of the analyzing process and were all present at the meetings and voiced their views. To improve transferability, a detailed explanation of the whole study process was provided, and participants were directly cited. The research findings were also shared with five others who were in similar situations as the study participants. However, they were not included in our study. They also confirmed that they had had comparable experiences to the participants.

## 3 Results

The current study included 22 female sex workers from Tehran, Iran's capital city. Table 2 displays the demographic information of the research participants.

**Table 2:** Demographic information of the participants in the study.

Characteristics		Frequency (%)
Age group	<25 years old	7 (32)
	25–40 years old	10 (46)
	>40 years old	5 (23)
Level of education	Elementary education	3 (14)
	Under high school education	6 (27)
	High school education	5 (23)
	Higher education	8 (36)
Age of first sexual intercourse	<15 years old	7 (32)
	16–30 years old	13 (59)
	>30 years old	2 (9)
Economic status	Low	11 (50)
	Average	7 (32)
	High	4 (18)
Marital status	Single	4 (18)
	Married	6 (27)
	Divorced or widowed	9 (41)
	Separated	3 (14)
Infecting with HIV OR Hepatitis	Yes	7 (32)
	No	15 (68)
The situation of drug addiction	Addicted	8 (36)
	Taking drugs for pleasure	11 (50)
Not a smoker	-	3 (14)

After evaluating the data, 5 primary categories and 19 subcategories (Table 3) were found, which will be discussed individually.

**Table 3:** The main categories and subcategories of sex work determinants.

Main Categories	Subcategories
Family instability	Family breakdown
	Seeking revenge for the husband's infidelity
	Failure to meet sexual needs in the family
Social pressure	Addiction
	Poverty
	Lack of proper context for marriage
Materialism	The dominance of materialistic values over moral values
	Daydreaming and aspiring to wealth
Insecurity in society	The bitter experience of first sexual intercourse
	Unwanted sex work
	Excessive demand for sex
	Problematic social learning
	Insecurity in occupational environments
Passive and inefficient personality	Lack of talents and job skills
	Failure to deal with issues effectively
	Low self-esteem
	Excessive attachment
	Diversity-seeking
	Narcissism

### 3.1 Family instability

The first element that contributes to the proclivity to participate in sex work refers to difficulties that arise in the hub of the family and compel the individual to engage in sex work.

#### 3.1.1 Family breakdown

Family issues have been found to be a significant contributing factor in a wide range of harmful behaviors. Participants in the current study mentioned living with a stepfather or stepmother, divorce and separation, and family quarrels and conflicts as inappropriate conditions that pushed them to engage in sex work.

*Participant # 3 said, “My parents got divorced and I had to live with my licentious stepfather. However, I moved away from there and engaged in sex work.”*

*Participant # 9 said, “My parents' constant bickering and arguing wore me down. As a result, I chose to leave and engage in sex work to support myself. ”*

#### 3.1.2 Seeking revenge for husband's infidelity

In some situations, women engage in extramarital affairs as a form of retaliation against their spouses for cheating on them. Women first feel fulfilled after exacting vengeance, but as the affairs progress, they become increasingly interested in sex work.

*Participant # 5 said, “We had been married for almost six years when I learned my spouse was cheating on me, which was difficult for me to accept. As a result, I resolved to exact my vengeance by having relationships with other men. I used to have sex without asking for money, but I gradually started doing it for money.”*

#### 3.1.3 Failure to meet sexual needs in the family

Failure to meet sexual needs in the family might expose couples to additional social consequences, such as sex work. That is, many women engage in extramarital affairs to satisfy their sexual urges, but become entangled in sex work as a result.

*Participant # 22 said, “My spouse did not satisfy me sexually. That's why I decided to find a lover to sleep with on occasion. My partner once asked me if I wanted to have an affair with his buddy as well. At first, I refused and became furious, but then he gave me a valuable present, which convinced me to accept his proposal. Later on, anytime they wanted me to sleep with them, they would buy me something. Then, over time, I got divorced and began doing sex work for money.”*

### 3.2 Social pressure

Sex work is a phenomenon that has developed in the center of society, and many female sex workers chose this career as a result of societal pressure.

#### 3.2.1 Addiction

In addition to being one of the consequences of sex work, addiction may also lead to sex work. As a result, there is a connection between addiction and sex work, and in many situations, women become addicted after engaging in sex work, and the sex work process accelerates after addiction since the person just wants to have access to drugs and sleep with anybody. Furthermore, addicted parents, particularly fathers, might force their wives and daughters to sleep with others in exchange for money.

*Participant # 12 said, “I didn't care about anything when I became an addict. I only had sex with my partner prior to addiction, but after addiction, I had to sleep with anybody who paid me. ”*

*Participant # 6 said, “When I was 16 years old, my addicted father took me to his friend's place to sleep with him.”*

#### 3.2.2 Poverty

Lack of financial resources is recognized as one of the primary motivators for women to participate in sex work. In other words, impoverished women typically engage in sex work to fulfill their most basic needs. Poverty forces people to seek aid from others, and because they are unable to reciprocate, they are compelled to sleep with them.

*Participant # 17 said, “We have been impoverished since I was a child, and my parents had to work incredibly hard. Then my father fell from a scaffold, and it was left to my mother to bring home the bacon, but she was barely able to maintain the family. As a result, they wanted us to marry as soon as possible. Later, my sister got engaged, and I had to raise funds for her dowry. As a result, I borrowed money from someone but was unable to repay it, leaving me with little alternative but to sleep with him.”*

#### 3.2.3 Lack of proper context for marriage

Another issue that motivates women to participate in sex work is a lack of marital prospects. When women's desires for marriage and sexual orientation are not satisfied through socially acceptable channels, they engage in sex employment. It should be mentioned, however, that their primary objective was not to be sex workers, but they unintentionally became so.

*Participant # 11 said, “I was unmarried and wanted to marry when I was 33, but I had no suitor. That is why I was forced to engage in sex work. ”*

### 3.3 Materialism

In today's Iranian society, materialism has intensified, and moral values have been devalued as a result. As a result, it encourages women to participate in sex employment.

#### 3.3.1 The dominance of materialistic values on moral values

Despite advancements in communication technology, ethical and traditional values have diminished in the modern world, while materialistic ideals have become more prevalent. As a result, women disregard societal moral standards, practices, and dominant ideals in order to satisfy their wants.

*Participant # 13 said, “It makes no difference to me how you make money. I believe you should be clever and make money, but I am only attempting to earn money by the beauty that God has bestowed upon me.”*

#### 3.3.2 Daydreaming and aspiring to wealth

According to the dominance of materialistic values over moral ones, some women regard sex work as a well-paid profession and a decent source of money, similar to other jobs, through which they may accomplish their goals.

*Participant # 3 said, “I didn't care how to get money. I just wanted to be wealthy. That is why I became a sex worker, and I believe our profession is not all that different from other jobs.”*

*Participant # 18 stated, "I've always wanted to get rich by any means possible, but I'm not particularly well-educated to make money." So I decided to become a sex worker in order to make a lot of money because I was attractive enough to attract a lot of men.”*

### 3.4 Insecurity in society

Women in Iran experience a form of social instability as a result of the country's social and cultural circumstances, which might lead to them engaging in sex work.

#### 3.4.1 The bitter experience of first sex

The first intercourse without the woman's permission is referred to as the first stage of forced sex work. Because virginity is seen as a symbol of purity and chastity in Iran, females who unintentionally engage in sexual encounters have their whole lives shattered, leading them to participate in sex work.

*Participant # 4 said, “Because I couldn't afford the rent, I had to sleep with my landlord. So I despised myself and wanted to commit suicide. After that, I didn't give a damn about anything. I was a staunch believer in God before sleeping with the landlord, and I always said my prayers on time, but after that, I felt like such a loser.”*

*Participant # 20 said, “I had my first intercourse when I was raped in an old neighborhood, but I kept it a secret from everyone. I couldn't marry when I grew up because I was afraid that everyone would find out and it would bring shame to the family. As a result, I tried suicide several times but never had the strength to do it. So I decided to depart without telling anyone. In addition, in exchange for a place to stay, I had to sleep with a man, and the rest is history.”*

#### 3.4.2 Unwanted sex work

Many women who are currently active in sex work were coerced or blackmailed into doing so, and many of them stated that they were forced or threatened to do so. Not to mention the numerous material and financial temptations from customers, as well as a lack of family and social support, which makes quitting sex work impossible.

*Participant # 2 said, “I never thought about having sex for money. I recall the first time I slept for money. I explained to myself why I was acting in this manner. Later on, I tried several times to leave, but it was too tough to do so, and I became more and more immersed in the sex business with each passing day.”*

*Participant # 11 said, “I've tried so many times to stop doing sex work, but I can't seem to stop myself. For example, I recall making a firm vow to cease doing sex work when one of my customers contacted me and offered me a large sum of money in exchange for accompanying him on a trip.”*

*Participant # 7 said, “I believe that anyone who has ever slept for money will find it difficult to stop. Even if she makes a sincere pledge never to do it again, others will not let her. For example, I frequently attempted to resign but was met with death threats from my clients. There was no one to aid me, and I was occasionally compelled to sleep with them even though I had no money.”*

#### 3.4.3 Excessive demand for sex

Women, particularly those who live alone, are subjected to a plethora of social ills and are subjected to a plethora of demands for unlawful sex, which may sometimes be deceptive and lead to sexual addiction to sex work. Society's perception of widowed and divorced women is inappropriate, and males regard it as their right to mistreat women.

*Participant # 8 said, “Everyone has turned into licentious wolves, and I have gotten several direct and indirect sex proposals from those around me.”*

*Participant # 16 said, “When I got divorced, many philanderers disturbed me, and even my boss's behavior changed. So I had to change my phone number, but it was futile because many others approached me and offered me sexual connections, which I found surprising.”*

#### 3.4.4 Problematic social learning

Learning how to do sex work is a significant stepping stone in the growth of sex work. For example, in certain places where there are street sex workers, their frequent journeys and seeing them conversing with people pique the interest of other women in their lifestyles and inspire them to become sex workers.

*Participant # 5 said, “I lived in a neighborhood with sex workers, which piqued my interest in what it was like to be a sex worker. I was once picked up from the side of the road by a vehicle that compelled me to engage in sex work.”*

#### 3.4.5 Insecurity in occupational environments

Despite the fact that women's employment has increased in recent decades, there are still numerous barriers to their presence, and many women feel uncomfortable at work in some situations. Because of the strong demand for illegal relationships in the workplace, women are compelled to abandon their employment, or, in other circumstances, they are forced to sleep with their elderly in order to keep their jobs and money.

*Participant # 14 said, “I never planned to be a sex worker, but it happened to me. I was a devout follower of God. However, my boss, coworkers, and others made too many sexual affair suggestions, leaving me with no choice but to resign from my job.”*

### 3.5 Passive and inefficient personality

A woman's personality, in addition to the concerns described above, gives additional grounds for enrolling in sex work.

#### 3.5.1 Lack of talents and job skills

Some respondents stated that they were forced to have sex for money due to a lack of abilities and employment skills.

*Participant # 10 said, “If I could have a respectable career, I would never do sex work." However, I have no idea how to do hairdressing as a career. That is why I had to work as a prostitute.”*

#### 3.5.2 Failure to deal with issues effectively

Another concern raised in the interviews was a lack of self-efficacy. When women have financial issues, they are unhappy, and some males approach those ladies and invite them to sleep with them. As a result, it entices them to participate in sex employment.

*Participant # 18 said, “When confronted with friendship suggestions from other guys, I lacked the willpower to reject them and was at a loss for what to do. So I agreed to their friendship recommendation, and the rest is history. ”*

#### 3.5.3 Low self-esteem

Another reason why women participate in sex work is a lack of self-esteem for altering their lives and attaining anything they wish.

*Participant # 3 said, “I've never been happy with my life, and I've always believed I was weaker than everyone else. That is why I never sought to alter things and instead chose to work as a sex worker.”*

#### 3.5.4 Excessive attachment

One of the difficulties raised by women in their interviews was their strong commitment to their emotional partners, which led them to give in to excessive requests from their spouses, such as sleeping with other people.

*Participant # 7 said, “I was head over heels in love with my partner and didn't want to lose him. So I was willing to go to any length to avoid losing him. He once invited me to sleep with one of his friends, but I refused at first. Later, he threatened to end our relationship if I did not agree to his request. Finally, I succumbed to his words and had sex with his friend and a few others.”*

#### 3.5.5 Diversity-seeking

Some sex workers stated that they sought diversity in their past relationships prior to marriage, which became a habit after marriage and caused them to disrupt their marital connections.

*Participant # 15 said, “I've always liked to put things to the test since I was a kid. For example, when I was in middle school, I had about 20 boyfriends, and after spending some time with one, I moved on to the next. When I got married, I couldn't stop the practice and began seeing many lovers at the same time, until my husband discovered my adultery and divorced me.”*

#### 3.5.6 Narcissism

Another issue that now concerns women and drives them to participate in sex work and act contrary to social standards is narcissism and attracting the attention of others. Although this curiosity is inherent in all women, it has paved the way for sex employment.

*Participant # 16 said, “I enjoy it when others are drawn to me. When I go out in public, I put on a lot of make-up and dress-up to attract people's attention. ”*

## 4 Discussion

The current research employed a qualitative method to investigate the reasons that predispose women to participating in sex work in Tehran.

The first reason for engaging in sex work was instability in the family structure. In other words, the living environment and family life of women form the foundation for sex work. Family breakup, living with stepfather and stepmother, and a lack of family support are all variables that lead to women's involvement in sex work. In addition, a lack of good connections between husband and wife, as well as a lack of fulfillment of women's sexual demands, might lead women to engage in sex work. Previous studies have identified family as one of the factors that drives women to engage in sex work [7, 23, 47, 48]. For example, in an Iranian research, the grounds for choosing sex work were the spouses' lack of emotional commitment and betrayal [10]. In an Iranian study conducted by Roshanfekr et al. (2015), the women said that one of the family's males coerced them into prostitution to make money [32]. Harassment of a wife following her husband's death was identified as a motivation for sex work in an Indian research [39].In another study done in Iran by Rostamzadeh et al. (2016), spouse harassment was highlighted as a key factor driving women to sex work. Other research has found that sexual unhappiness in women is one of the reasons they engage in sex work [49, 50, 51]. According to Rostamzadeh et al. (2016), one of the reasons for having sexual connections with other men in Iran is the lack of warmth and closeness between the spouses in sexual interactions [7]. Women find safety and security in their families. However, if it fails to perform its tasks correctly, it will cause deviations in the family. According to the majority of the women in the research, they grew up in a dysfunctional household where their spouses deceived them and their sexual demands were unsatisfied.

The current study's findings revealed that many women were enamored with luxury, and in their materialistic perspectives, sex work was viewed as a source of income and one of the simplest and finest ways to achieve their goals. Similarly, in an Iranian research, Karamouzian et al. (2016) found that materialism, or concern for money, was a major factor in pushing women into sex work [37]. Other studies have found that sex work is the easiest and most cost-effective method to make money [26, 52, 53]. Cultural interactions through the media and other channels have altered people's perceptions in Iran. Nowadays, materialism is the dominant characteristic of civilization, and everyone is seeking for a way to get wealthy. Meanwhile, other women characterized sex work as the highest-paying career in which they could work for a few years to attain their ambitions, which is why they opted to work as sex workers.

Other major reasons for getting into sex work were societal pressure and difficulties such as addiction, poverty, and marital conflicts. Similarly, poverty [7, 23, 40, 52, 54, 55, 56, 57] and addiction [32, 58] have been identified as major predictors of sex work. McMillan and Worth (2019) discovered that sex work is undertaken in Pacific nations owing to economic need [59]. According to McMillan and Worth (2019) and Foley (2019) research, one of the causes of women entering sex work was financial necessity induced by unemployment [59, 60]. In recent years, the challenges of employment and financial necessity have become even more pressing in Iran. Women's engagement in sex work increases under tough economic situations and material needs.

One of the intriguing and novel findings of this study that has not been highlighted in previous studies is that the inability of women to marry on time leads them to participate in sex work. Many young people have been unable to marry in recent years as a result of changes in the social and cultural framework, as well as economic issues. This issue leads young people to satisfy their sexual demands without marrying. As a result, sex work is becoming more prevalent in society.

Insecurity in society was the fourth reason for sex work. One of the more alarming findings of this study was the unpleasant experience of first sexual intercourse. This problem has been discovered in several studies, and a history of rape as a kid is frequently cited as one of the causes for a proclivity of sex work [23, 48, 53, 61, 62]. Because sexual intercourse without marriage is considered offensive and unforgivable in Iran, girls who are raped in their childhood or adolescence face many difficulties in their lives; on the one hand, they cannot complain against the wrongdoer because revealing rape can affect the victims' entire future and cause them to be rejected in society. Furthermore, for fear of being exposed, the victim will not visit a doctor or a psychologist. Furthermore, because she has lost her virginity, she can no longer consider marriage because fewer individuals are willing to marry such a person. This problem drives women into the sex business.

Excessive sex demand was another factor that pushed women into sex work, and it was consistent with prior studies in this area [60, 63, 64]. Farley and Butler (2012) revealed that men's high sex needs are a deciding factor in sex work [65]. Sex work is a two-way street that needs both men and women to perform, with males typically wanting more. Sex employment is expected to grow significantly as men's need for sex grows in culture. However, in most studies, preventative and initiatives, men's roles and contributions are frequently overlooked.

Understanding sex work in the context of social life, as well as living in regions where sex workers reside, were two key results of the current study. According to Yavari and Haji Dehabadi (2018), the presence of corrupt neighborhoods in cities in Iran is a significant influence on sex work [66]. This issue, particularly among younger women and girls, might lead to deviations and sex work, since sex workers' frequent commutes and seeing them conversing with strangers in expensive automobiles piques the interest of other women in their lives. As a result, it inspires other women to learn how to operate as sex workers. According to Roe-Sepowitz (2012) in the United States, some sex workers had poor socioeconomic levels and lived in areas where sex work was widespread [30].

Another outcome of the study was workplace insecurity. The women in the current study stated that they did not feel secure at work and were suggested to have affairs, which drove them to resign or accept offers for economic reasons or threats. As a result, it had a negative impact on both situations. One of the major reasons for sex work in the Philippines was the employers' efforts to fool the opposite gender [54]. Because of the growing employment of women in several industries, occupational instability is concerning. It should be emphasized that a substantial number of women may have no choice but to accept the offers of their coworkers due to financial constraints.

Another major factor in sex work is women's poor self-efficacy. This discovery adds to earlier studies showing that, in addition to the environment and culture, some personality characteristics might put women at a higher risk of engaging in sex work. Many sex workers are persuaded to have sex for money due to a lack of abilities and professional skills. In other words, if individuals had high levels of self-confidence and self-efficacy, they were more likely to live healthy lifestyles. According to research, the motivations for engaging in sex work include a lack of jobs and career possibilities, as well as a need for money for themselves and their families [32, 47, 60, 67]. In a research conducted by Liu (2012), women claimed that they were performing low-paying occupations owing to a lack of education and skills prior to engaging in sex work [40]. In a research conducted by Farley and Butler (2012), women stated that if they were hired and taught how to obtain employment, they would be able to live healthy lives and stop doing sex work [65]. In a research conducted in the Netherlands, women indicated decreased self-confidence in their capacity to obtain alternative occupations as a cause for beginning and continuing sex work [8]. According to the findings of our study, low self-esteem and excessive reliance on emotional relationships predispose women to sex work. In a research conducted in Pakistan, women also [23]. Because of Iran's social, cultural, and patriarchal circumstances, most women lack confidence in dealing with difficulties and problems. As a result, many engage in sex work since they lack the necessary knowledge and abilities to perform other professions.

One of the issues confronting sex workers in Iran is a lack of support and help from social and legal frameworks. As a result, they have limited access to social services and health care. Sex workers have limited access to health services like HIV testing, contraception, and counseling, as well as no special housing and little legal protection. As a result, they are vulnerable to a wide range of dangers. According to the findings of a study conducted in Iran by Yoosefi Lebni et al. (2020), sex workers do not have social or legal protection and hence experience a great deal of harm [41]. Shushtari et al. (2018) discovered a link between social support and a decrease in risky sexual behaviors, condom usage, and, as a result, a decrease in the risk of HIV and other infections [68]. According to a research conducted by Asadi-Aliabadi et al. (2018) in Iran, sex workers are less likely to get healthcare services and are less inclined to undergo medical testing and receive health care owing to limitations and pressures [69]. When individuals are harassed and their personal and societal rights are infringed, this lack of support becomes much more obvious. When people go to organizations to complain, they may be judged guilty. Social support for these women should be in line with legal, cultural, and social aid, and chances for empowerment should be provided. Improving the health of sex workers necessitates a shift in policymakers' and non-governmental organizations' attitudes, new regulations, social support (e.g., contraceptive education and tools, proper nutrition, and safe places to live), and the establishment of free counseling centers to empower these women.

## 5 Limitations

Since sex work is prohibited in Iran, it is done behind closed doors. The most significant drawback of this study was the difficulty in gaining access to the study group due to a lack of reliable data or information regarding the number and location of sex workers. Another drawback of this study was obtaining consent from female sex workers to participate in the study. The researchers were able to obtain their consent by continuous follow-up and clarification of the whole interview procedure, as well as how the results would be published. The study was only done in Tehran, and the number of participants was small. Other studies using qualitative and quantitative methodology, as well as a large number of samples, should be done in other Iranian cities. Furthermore, since the findings revealed that males are one of the primary reasons why women engage in sex work, it is important for men who have relationships with sex workers' families to participate in studies in order to have a better understanding of the issue in future studies.

## 6 Conclusion

The findings indicate that sex work in Iran is influenced by a variety of factors, including family structure, societal pressure, social instability, consumerism, and a passive and ineffectual mentality. So, social damage may be avoided by improving women's social status, boosting economic support, strengthening intra-family connections, raising self-esteem and self-efficacy, learning how to deal with life's issues, and making society safer, particularly in the workplace. Future research might look at the reasons why women engage in prostitution in different Iranian cities, as well as the cultural and geographical contrasts and similarities in the setting of prostitution. They can also investigate these women's difficulties, such as violence against them.

## Declarations

### Author contribution statement

Javad Yoosefi Lebni: Conceived and designed the experiments; Wrote the paper.

Seyed Fahim Irandoost: Conceived and designed the experiments; Analyzed and interpreted the data; Wrote the paper.

Arash Ziapour, Bahar Khosravi: Performed the experiments; Contributed reagents, materials, analysis tools or data.

Ali Akbar Dehghan: Analyzed and interpreted the data; Contributed reagents, materials, analysis tools or data.

Nafiul Mehedi: Analyzed and interpreted the data.

### Funding statement

This work was supported by 10.13039/501100005317Kermanshah University of Medical Sciences.

### Data availability statement

The authors do not have permission to share data.

### Declaration of interests statement

The authors declare no conflict of interest.

### Additional information

No additional information is available for this paper.
